# Worldwide prevalence of mother-infant skin-to-skin contact after vaginal birth: A systematic review

**DOI:** 10.1371/journal.pone.0205696

**Published:** 2018-10-31

**Authors:** Nawal Abdulghani, Kristina Edvardsson, Lisa H. Amir

**Affiliations:** 1 Judith Lumley Centre, La Trobe University, Melbourne, Australia; 2 Faculty of Nursing, Umm Al-Qura University, Makkah, Saudi Arabia; TNO, NETHERLANDS

## Abstract

**Background:**

Despite the World Health Organization’s (WHO) recommendation for immediate skin-to-skin contact (SSC) after birth, separation of mothers and infants seems to be common practice in many hospitals. It is unknown how common the practice of SSC is worldwide. Therefore, we aimed to determine the reported prevalence of SSC for healthy mothers and infants immediately after normal birth.

**Methods:**

We systematically searched CINAHL, Medline, ProQuest Central, PubMed and the Cochrane Library for articles published between January 2007 and October 2017 using the keywords "kangaroo care" or "skin to skin contact" or "breastfeeding initiation" or "breast crawl" or "maternal infant contact" or "maternal newborn contact" or "baby friendly hospital initiative" or "ten steps for successful breastfeeding”.

**Results:**

After an initial screening of 5266 records, 84 full text articles were assessed for eligibility, and 35 of these met the inclusion criteria. The studies were from 28 countries representing all six WHO world regions. There was a wide range in the practice of SSC for mother-infant dyads around the world: from 1% to 98%. Only 15 studies clearly defined SSC. Most of the studies were from high-income countries, and these reported higher rates of SSC than studies from low and middle-income countries.

**Conclusion:**

There was a great heterogeneity in the definition of SSC as well as study designs, which makes cross-county comparison difficult. National studies reporting SSC rates are lacking. Future studies and guidelines to enhance SSC practice should include a standardised set of indicators and measurement tools that document SSC starting time and duration of SSC.

## Introduction

Skin-to-skin contact (SSC) is defined as placing the naked baby on the mother’s bare abdomen or chest immediately or less than 10 minutes after birth or soon afterwards [1]. The World Health Organization (WHO) recommends the practice of SSC for at least one hour after birth, and health care providers should encourage women to recognise when their babies are ready to breastfeed and offer help if needed [1]. Evidence about the benefits of SSC have been compiled for a Cochrane Review and meta-analysis, and these indicate that mothers who had SSC were more likely to be breastfeeding at one to four months after birth, had longer breastfeeding, exclusively breastfeed from hospital discharge to six months after birth and infants who received SSC had higher stability of the cardio-respiratory system, and higher blood glucose levels [2]. Furthermore, protocols and position statements from the Academy of Breastfeeding Medicine (ABM) [3], American Academy of Pediatrics (AAP) [4] and Association for Women’s Health, Obstetric and Neonatal Nurses (AWHONN) [5], WHO and United Nations International Children's Emergency Fund (UNICEF) [6] clearly support SSC practices. However, there are no agreed set of standardised universal practice guidelines for SSC care at present.

Despite recommendation for immediate, continuous and uninterrupted SSC, separation of mothers and infants is common in many hospitals and infants are often placed in cots or under warmers [7]. It is not known how common the practice of SSC is worldwide. Therefore, it is timely to identify the prevalence of SSC for mothers and newborn infants. The aim of this review is to systematically describe the reported prevalence of SSC for healthy mothers and infants immediately after vaginal birth, from data published between 2007 and 2017 worldwide, in order to estimate the current practice of SSC.

## Methods

### Inclusion and exclusion criteria

Pre-defined inclusion and exclusion criteria were set for the review, including type of participants, type of publication and study designs, and context of studies. The inclusion criteria were:

#### Type of participants

- Adult women aged ≥18 years.
- Normal birth including instrumental assisted birth.
- Healthy pregnancy.
- Full-term newborn infant.
- Healthy baby not requiring any resuscitation or transfer to Neonatal Intensive Care Unit (NICU).

#### Type of publication and study design

- Original research: Randomised Control Trial (RCT) (only participants in the control group were included), cohort studies, observational and cross-sectional studies.
- Publication date between January 2007 and October 2017.
- Peer-reviewed journal.
- Published in any language.
- Sample size > 100 participants.

#### Context

- Worldwide.

The exclusion criteria were:

#### Type of participants

- Studies focused on women with medical complication such as diabetes, high blood pressure, or HIV/ AIDS.
- Multiple gestation.
- Subgroups of the population not representative of the general population at large, for example, adolescent mothers or particular ethnic groups.
- Preterm babies born before 37 completed weeks of gestation.
- Low-birth-weight baby less than 2500 grams.
- Babies with deformity or health conditions required admission to NICU.

#### Type of publication and study design

- Review articles.
- Letters to the editor, conference proceedings, and abstracts.
- Studies measuring SSC from an administrative level.
- Low quality studies according to Joanna Briggs Institute (JBI) assessment of methodological quality checklist [8] with more than three questions rated No or Unclear.

### Study outcomes

The primary outcome for this review is the prevalence of skin-to-skin contact for healthy newborn infants > 37 weeks of gestation after normal birth. Secondary outcomes are starting time for SSC and duration of SSC.

### Search strategy

**Fig 1:**
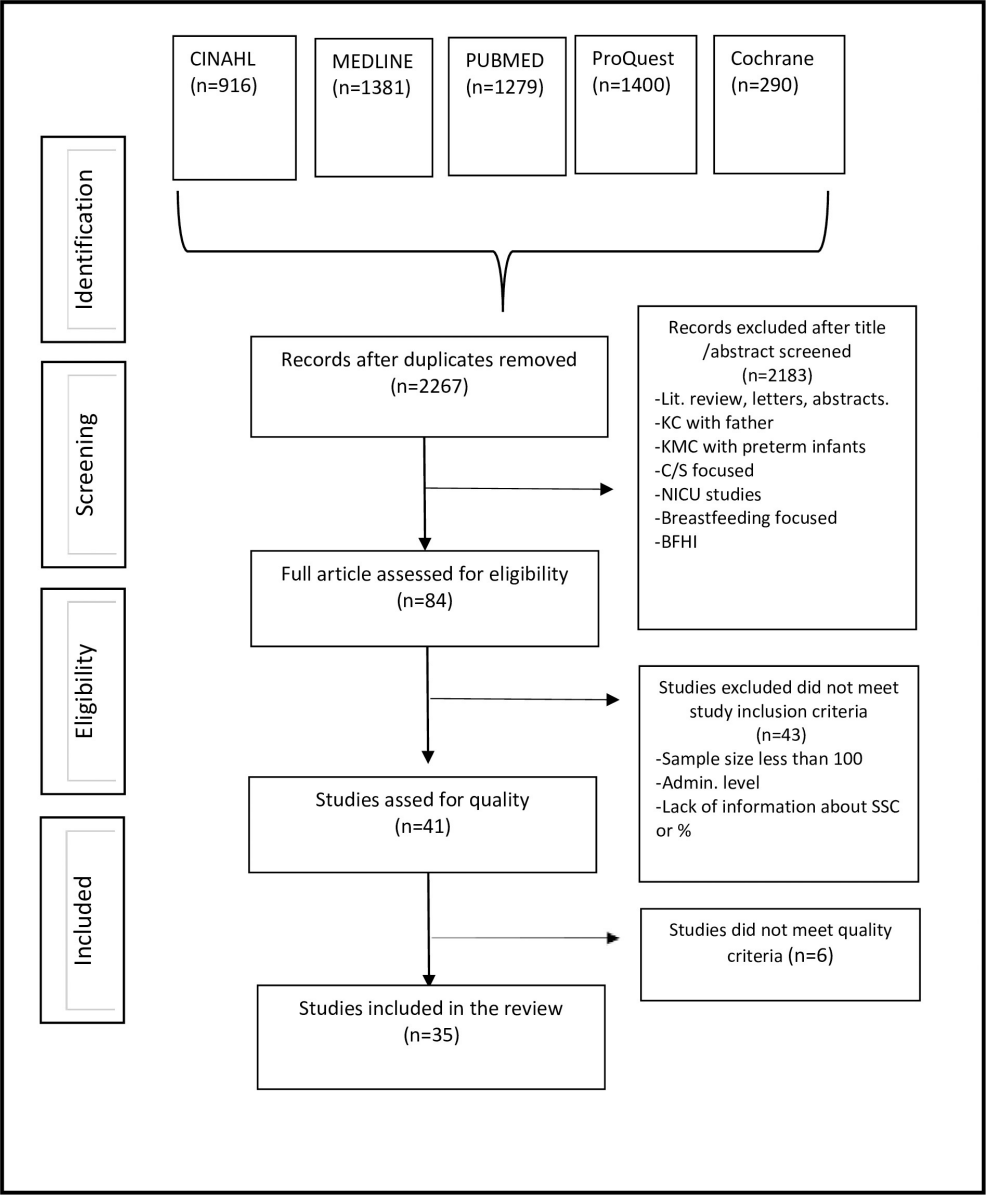
PRISMA flowchart. Lit: Literature review, C/S: Caesarean Section, KC: Kangaroo care, NICU: Neonatal Intensive Care Unit, BFHI: baby friendly hospital initiative, Admin: Administration.

The following databases were searched: CINAHL, Medline, ProQuest Central, PubMed and the Cochrane Library using the keywords "kangaroo care" or "skin to skin contact" or "breastfeeding initiation" or "breast crawl" or "maternal infant contact" or "maternal newborn contact" or "baby friendly hospital initiative" or "ten steps for successful breastfeeding”. A full electronic search strategy for the CINAHL database is presented in (S1 Table). Articles published from January 1, 2007 to October 20, 2017 were reviewed and assessed for eligibility. This time frame was selected to estimate the current reported prevalence of SSC worldwide. The PRISMA-P 2015 protocol for systematic reviews and meta-analyses was used to guide this review (Fig 1) [9].

### Search outcomes

As shown in the PRISMA flowchart (Fig 1), the literature search resulted in a total of 5266 records. After removal of duplicates, 2267 records remained. Following screening of titles and abstracts, a total of 84 full articles were assessed further for eligibility. Forty-three studies were excluded because the sample size were less than 100, the practice of SSC were reported from administrative level or information about SSC were insufficient. After rating the quality of the remaining 41 studies using the JBI assessment of methodological quality checklist tool [8], six studies were excluded because of insufficient clarity. Thirty-five studies remained and were included in the review.

### Assessment of methodological quality

The JBI critical appraisal checklist was chosen because it is designed to appraise studies reporting prevalence data [8]. The JBI tool consist of ten questions about the study sample adequacy and appropriateness (questions 1, 2, 3 and 4), the validity and reliability of data collection methods (questions 5, 6 and 7), and the analysis of outcome measurement and response rate (questions 8 and 9). The appraisal process was independently undertaken by two reviewers and any disagreement was resolved by a third reviewer. Reviewers looked for the strengths and weaknesses, and validity and biases of each study by answering a standardised ‘Yes, No, Unclear or Not applicable’. If any study had more than three No or Unclear responses, the reviewers excluded the study. Forty-one studies were assessed for their quality; six studies were excluded because of lack description of methods or ambiguous data. The quality indicator for the 41 studies are presented in S2 Table, Joanna Briggs Institute (JBI) assessment of methodological quality tool check list.

### Data extraction

Data abstracted included (1) year of publication; (2) country of study; (3) aim of the study; (4) study design; (5) numbers and characteristics of participants; (6) SSC definition; (7) proportion of SSC; (8) starting time of SSC; (9) and duration of SSC were available. In some studies, the number of and percentage of SSC practice were reported for both vaginal and caesarean section birth combined. The first author therefore contacted the author of each of these studies to request data about vaginal births only. In total, 33 authors were contacted regarding data presented in 30 studies. Three reminders were sent, and if no response was received, the study was excluded if the quality indicator using the JBI critical appraisal checklist tool was not met. The literature search included all languages, and two studies in French and Finnish were included. Accredited translators assisted to translate these two papers.

### Data synthesis

The total population from all 35 studies was 429,222 mothers or hospital records about SSC practice, whether mothers had SSC after normal birth or not. Due to the studies heterogeneity, it was not possible to perform meta-analyses. Narrative synthesis with tabulation of studies and graphical explanation were used to summarise data. The review reports the proportion of SSC immediately after normal birth, and the starting time and duration of SSC where available for research published during the period between 2007 and 2017.

## Results

### Description of the included studies and context

The included studies were diverse in methods and focus. Only three studies focused specifically on SSC practices after birth [10–12]. Six studies focused on breastfeeding and SSC [13–18]. The remaining studies focused on other aspects related to maternal care during and after birth, breastfeeding and evaluation of the Baby Friendly Hospital Initiative (BFHI).

The included studies represented 28 low, middle and high-income countries: Australia (n = 5), Brazil (n = 2), Cambodia (n = 1), Canada (n = 2), Croatia (n = 1), Denmark (n = 1), Ethiopia (n = 1), Finland (n = 1), France (n = 1), India (n = 1), Italy (n = 1), Japan (n = 1), Mexico (n = 1), Nepal (n = 1), New Zealand (n = 1), Philippines (n = 1), Singapore (n = 1), Sri Lanka (n = 1), South Korea (n = 1), Spain (n = 2), Switzerland (n = 1), Taiwan (n = 1), Tanzania (n = 1), Tunisia (n = 1), United Kingdom (n = 1), United States of America (n = 1), Yemen (n = 1), and a multisite study covered Bangladesh, India, and Nepal.

### Definition of SSC practice used in the studies

Although the practice of SSC is defined by the WHO, the definition varied among the included studies. Some studies reported SSC practice without defining it, and only 15 studies defined the practice of SSC. The definitions of SSC reported in the included studies are presented in Table 1. Most of the definitions articulated in included studies were with emphasis on the newborn infant with or without clothes, newborn infant position on the mother’s chest or abdomen and duration of infant-mother SSC. Four studies defined SSC as the placing of the naked baby on the mother’s chest or abdomen [14, 16, 18, 19], whereas another study allowed the baby to wear cap, diaper and socks [13]. Two studies mentioned in their definition of SSC that both the mother and the infant need to be covered with a warm blanket or dry towel to stabilise the infant [17, 18] Four studies explained in their definitions of SSC, that the newborn infant’s position should be placed prone on the mother’s bare chest or abdomen [16, 18–20]. Three other studies stated the time that the newborn infant stayed SSC with the mother for 30 minutes [16], first hour [11] or two hours [21].

**Table 1:** Skin-to-skin contact definitions in included studies where defined (alphabetical) by author/s name.

Author/s	SSC definition
**Andersson et al (2016) [21]**	“….close contact between the mother and newborn infant established within the first 2 hours after the child born”p.598
**Brodribb et al (2013) [22]**	“…the first contact”p.686
**Callaghan-Koru et al (2016) [13]**	“The baby is naked with the exception of cap, diaper and socks” p.e569
**Chalmers et al (2010) [23]**	“Mother was naked at first contact with baby” p.47
**Hongo et al (2015) [19]**	“Mother holding the baby prone against her chest within 5 min of birth, sustaining that position for more than 30 min, and being offered help with breastfeeding by staff” p.1245
**Chiou et al (2014) [14]**	“…baby was put on her chest immediately after a vaginal birth or within 1 hour after a caesarean delivery” p.34
**Fritz et al (2017) [24]**	“…immediate contact between mother and child after birth” p.6
**Haiek (2012) [25]**	“Naked baby on mother’s naked body” p.896
**Kalmakoff et al (2017) [26]**	“….place the baby skin-to-skin within 5 min of birth for at least one hour” regardless of birth type”p.2
**Kim (2016) [16]**	“Placing of the naked baby prone on the mother’s bare chest within 30 minutes after delivery” p.3
**Lau et al (2017) [17]**	“Placement of naked infants on mothers' bare skin; the exposed side or back of infants is covered by dry towels or blankets”p.2
**Martinez-Galiano and Delgado Rodriguez (2014) [11]**	“To place new-born on the mother’s lap after delivery for the first hour of life”p.720
**Redshaw et al (2014) [12]**	“Was your baby straight on your skin and not wrapped, dressed or in a nappy?”p.e179
**Saxton et al (2015) [18]**	“The naked healthy newborn baby is placed prone on the mother’s bare abdomen/ chest immediately after birth in a position where the baby has ready access to the maternal nipple. Both mother and baby should be covered with a warmed blanket”p.1111
**Suarez-Cortes et al (2015)[20]**	“Placement of the infant in the prone position in direct contact with the mother”p.522

### Primary outcome: The global prevalence of skin-to-skin care

Table 2 presents the 35 included studies arranged according to the six WHO world regions. Our search of the databases revealed that the highest number of studies were from The Western Pacific Region (n = 12), followed by European Region (n = 9), Region of the Americas (n = 6), South-East Asia Region (n = 4), African Region (n = 2) and Eastern Mediterranean Region (n = 2). It is important to note that the included studies may not be representative of each country. These studies were the most recent resource that reported the practice of SSC. Most of the included studies have indicated that women reported the practice of SSC. Several studies reported data from midwifery/ perinatal registration [11, 16, 18, 20, 21, 26–29].

**Table 2:** Summary of included studies categorised by WHO regions.

Continent/ Country	Author/s	Design	Sample	Methods of data collection	Proportion of SSC % (n/N^a^)	Type of birth % (n)	
						Vaginal	C-section
**African Region**							
**Ethiopia**	Callaghan-Koru et al (2016) [13]	Intervention study	218 women	Pre-intervention baseline survey, collected between 1–7 months after birth, about SSC, breastfeeding and other newborn care practices	**9.2%** **(20/217)**	NG	
**Tanzania**	Penfold et al (2010) [42]	CSS & RS	22,243 women ^**c**^	Questionnaire given at home visits, about newborn care practices ^DCNS^	**Less than 1% (144/22,243)** ^**c**^	NG	
**Region of Americas**							
**Brazil**	Bladisserotto et al (2016) [37]	RS	4,156 women	National survey “*Birth in Brazil*”, electronic survey administered face-to-face to women within the first 24 months	**34.1% (1,413/4,145)**	73.5%)3,055( Ins.D. 4.2% (173)	22.3% (928)
**Brazil**	Moreira et al (2014) [38]	RS	18,639 women	National survey “*Birth in Brazil*”, electronic survey administered face-to-face to women within the first 24 months	**41.9% (3,799/9,082)**	69.6% (6,324) ^**c**^	72.3% (6,911) ^**c**^
**Canada**	Chalmers et al (2010) [23]	National survey	6,421 women	Computer-assisted telephone interviews between 5–10 months after birth about labor, birth, mother-infant contact, and breastfeeding. experiences	**50.2%** **(2,811/5,600)**	73.7% (4,734)	26.2% (1,687)
**Canada**	Haiek (2012) [25]	Mixed method	176 women	Telephone interview at an average of two months after birth by using BFHI-40 Assessment Tool	**81%** **(121/150)**	100% (150)	
**Mexico**	Fritz et al (2017) [24]	RCT	641 women	Pre-training program baseline data from birth observations about birth practices	**9%** **(29/323)**	100% (323)	
**USA.**	Bramson et al (2010) [10]	PR cohort	21,842 women	Survey and interview after birth about SSC and breastfeeding	**17.3% (3,749/21,842)**	69.8%)15,876 (^**c**^	
**South-East Asia Region**							
**India**	Upadhyay et al (2012) [41]	CSS	415 women	Interview with mothers at 1 to 2 months at home visit about cord care, breastfeeding, thermal care, baby handling and health care seeking	**14.5%** **(60/415)**	94.7% (393)	
**Nepal**	Cederfeldt et al (2016) [44]	CSS	164 women	Self-administered questionnaire completed by mothers after birth at labour ward about intrapartum care	**16.5%** **(27/164)**	75% (124)	
**Sri Lanka**	Senarath et al (2007) [39]	Interventional study	892 women	Pre-intervention Interview at time of hospital discharge about newborn care practices	**50.4%** **(185/367)**		
**Nepal Bangladesh and India**	Crowe et al (2015) [40]	RCT	8,939 births records from Eastern India (E.I) and 27,553 births records from Bangladesh (B)	Survey after birth about newborn care practices ^DCNS^	**E.I = 15% (1,341/8,939)** **B = 30% (8,266/27,553)**		
**European Region**							
**Croatia**	Zakarjia-Grovic et al (2017) [34]	PR, longitudinal study	1,115 women	Survey completed at postnatal ward about BFHI Step 3	**97.8%** **(573/586)** ^**b**^	100% (586)	
**Denmark**	Andersson et al (2016) [21]	Nationwide, RT	269,597 births records	Data from Danish Medical Birth Registry	**95.9% (36,046/37,584)** ^**b**^		
**Finland**	Hakala et al (2017) [15]	CSS	111 mothers	Questionnaire completed at birth room about SSC	**89% (99/111)**	100% (111)	
**France**	Callendret et al (2015) [29]	PR Cohort	993 mother-child pairs	Observation at birth about BFHI practices	**64.9%** **(612/942)**	NG	15.4% (151)
**Italy**	Lauria et al (2016) [35]	Population based follow-up study	4,500 women	Interviews with women after giving birth about breastfeeding	**80.4% (3,620/4,500)** ^**b**^		
**Spain**	Martinez-Galiano and Delgado-Rodriguez (2014a) [11]	Observational study	520 primiparous women	Clinical charts data at birth about birth practices	**29.1%** **(113/389)** ^**b**^	74.34% (84), Ins.D. 7.08% (8)	18.58% (21)
**Spain**	Suarez-Cortes et al (2015) [20]	CSS	9,303 births records	Data from hospital records about the current situation of the delivery and birth plan ^DCNS^	**27.4% (2,549/9,303)**	73.8% Ins.D. 23.9%	2.3%
**Switzerland**	Gubler et al (2013) [27]	RS study	1,893 birth records	Data were divided according to three parameters maternal, infant and postpartum. ^DCNS^	**95.4% (1,806/1,893)**	53.8% (1019) Ins.D. 11.1% (211)	35% (663)
**UK**	Macfarlane et al (2014) [36]	Intervention study	259 women	Telephone surveys six weeks after birth about mother’s experience of midwifery care	**64.4%** **(65/101)**	100% (101)	
**Eastern Mediterranean Region**							
**Tunisia**	Bouanene et al (2010) [43]	CSS	354 women	Interviews at six months child vaccination about the knowledge and practices of breastfeeding	**63.8%** **(226/354)**		
**Yemen**	Kemp et al (2010) [45]	Qualitative	220 women	Questionnaire after birth about women’s authority at birth.	**7.8%** **(17/220)**		
**Western Pacific Region**							
**Australia** **QLD**	Brodribb et al (2013) [22]	RT Cohort	6,752 women	*2010 Having a Baby QLD Survey* posted at 4 months postpartum	**72.2% (4,874/6,752)**	65.5% (4,422)	34.5% (2,330)
**Australia** **QLD**	Keemer (2013) [30]	RS	128 women	*Breastfeeding Self-Efficacy Scale*- short form (BSES-SF) in day 7 after birth	**93% (119/128)**	41% (53) Ins.D. 12% (15)	47% (60)
**Australia** **NSW**	Ogbo et al (2016) [28]	RS	17,564 birth records	Perinatal data on all live births in 2014	**88.3% (11,489/13,003)**	77.0% (10,017) Ins.D. 11.3% (1472)	11.6% (1514)
**Australia** **QLD**	Redshaw et al (2014) [12]	Secondary analysis of survey	4,574 women	*2010 Having a Baby QLD Survey* posted at 4 months postpartum	**93% (2,979/3,189)**	100% (3,189)	
**Australia NSW**	Saxton et al (2015) [18]	RS	7,548 birth records	Audit via the electronic data base ObstetriX	**94.5%** **(7,133/7,548)**	77.6% (5,855)	
**Cambodia**	Sandin-Bojo et al (2012) [32]	CSS	177 women	Survey used the Bologna Score collected by midwives after each birth about birth care	**74%** **(107/144)**	100% (144)	
**Japan**	Hongo et al (2015) [19]	CSS	363 breastfeeding mothers	Self- administered questionnaires at infant’s 4-month health checkup about mother’s breastfeeding satisfaction	**20%** **(71/363)**	85% (310)	
**New Zealand**	Kalmakoff et al (2017) [26]	RS	1,530 birth records	Maternity Plus (2011) electronic data collection system	**69.5% (1,063/1,530)** ^**b**^	70.5% (1,080)	29.4% (450)
**Philippines**	Sobel et al (2011) [33]	Observational study	481 mothers–baby pairs	Intrapartum assessment tool about newborn care practices	**9.6%** **(46/481)**	76.3% (367) Ins.D. 1.5% (7)	C/S 22.2% (107)
**South Korea**	Kim (2016) [16]	RS	366 women	Medical record audit about factors influenced breastfeeding	**76.3%** **(184/241)**	70.9% (171)	
**Singapore**	Lau et al (2017) [17]	CSS	915 women	Structured questionnaire completed in delivery ward to assess intrapartum and SSC in relation to breastfeeding	**91.9%** **(677/737)**	80.5% (737)	19.4% (178)
**Taiwan**	Chiou et al (2014) [14]	National surveys	12,455 women	Telephone interview with women using structured questionnaire at 6 months postpartum about skin-to-skin contact, rooming-in, and breastfeeding:	**63.8% (4,995/7,828)**^**b**^	63.5% (7,911)	

^a^ Denominator is the number of women/records used in our calculation.

^b^ Data provided by author/s

^c^ Data as presented in the study

DCNS: Data Collection time Not Stated, CSS: Cross-sectional study, NG: Not given, RS: Retrospective study, RCT: Randomised Control Trial, PR: Prospective Study, Ins. D: Instrumental delivery, BFHI: Baby Friendly Hospital Initiative

#### Western Pacific Region

The Western Pacific Region provided most evidence for this review with 12 included studies. These studies varied in both design and location. Five studies were conducted in Australia and one study was conducted each in the following countries: Cambodia, Japan, New Zealand, Philippines, South Korea, Singapore, and Taiwan. In Australia, three studies were conducted in Queensland [12, 22, 30] and two studies in New South Wales [18, 28]. Two of these studies conducted in Queensland used the *2010 Having a Baby in Queensland Survey* [31]. The proportion of SSC in Queensland ranged from 72% to 93%, and in New South Wales from 88% to 95%. Therefore, the practice of SSC in these Australian studies was estimated to be between 72% and 95%.

A recent study conducted in Singapore determined that 92% of mothers had immediate SSC after normal birth [17]. In South Korea, a retrospective study reported that 76% mothers experienced SSC for the first 30 minutes after normal and caesarean section births (the normal birth rate was in this study 71%) [16]. A cross-sectional study conducted in Cambodia reported that 74% of mothers had SSC with their infants for at least 30 minutes [32].

In New Zealand, a retrospective study examined 1530 electronic records of healthy term infants and their mothers in 2011 to identify the predictors for supplementation for breastfed babies in a Baby Friendly Hospital. It was found that 69% had SSC after normal birth [26]. A national study conducted in Taiwan involving 12,455 women, examined the practice of early SSC and rooming in and their association with breastfeeding in 2004 and 2011. Data from 2011 was included only because it reflects the most recent prevalence estimate and represents the same population in Taiwan; the rate of SSC after vaginal birth was 64% in that year [14]. The remaining two studies in the Western Pacific Region were conducted in Japan [19], and the Philippines [33], and they reported a low proportion of SSC with 20% among Japanese mothers (reported at four months health checkups), and 10% among mothers in the Philippines.

#### European Region

In Europe, the included studies were conducted in eight countries including Croatia, Denmark, Finland, France, Italy, Spain, Switzerland and the UK. A recent study in Croatia identified hospital practices and breastfeeding rates before and after BFHI implementation [34]. A total of 773 mothers were included in the pre-BFHI group. In this study the data of SSC and vaginal birth data were reported separately [34]. The author of the study provided information following a request from the authors of this review, which indicated that 98% of mothers had SSC after vaginal births among the pre-BFHI group.

A national study in Denmark aimed to measure the quality of care provided during births [21]. Data on women and newborns representing 269,597 births was obtained from the Danish Medical Birth Registry, showing that 96% of women had SSC after normal birth [21]. In a cross-sectional study that described breastfeeding initiation and SSC implementation in eight maternity hospitals in Finland [15], 111 mothers were surveyed about their experience of breastfeeding and SSC. In this study, data of SSC and vaginal birth were reported separately. The authors of the study estimated that SSC among women who had a vaginal birth was 89% [15]. In a population-based study conducted in Italy, 80% of women reported at discharge that they had SSC and initiated breastfeeding within the 1^st^ hour postpartum [35].

Even though the sample sizes and contexts of the studies differed, two studies conducted in France and UK reported similar percentage of SSC at 64% [29, 36]. In Switzerland, a study analysed postpartum parameters including time to first SSC, time of first suckling and length of rooming-in [27]. Within the first hour, a total of 95% of mothers experienced SSC [27]. In two Spanish studies where practices after birth were observed [11, 20], the proportion of SSC were similar at 27% and 29%, respectively [11, 20].

#### Region of the Americas

In North America, two studies were conducted in Canada, one in the USA and one in Mexico. A national study in Canada surveyed 5600 women in relation to the development of a computer-based tool that measures policies and practices outlined in the BFHI. Fifty percent of women in this study reported that they had SSC experience in 2010 [23]. The second study from Canada reported a higher percentage of SSC practice at 81% in 2012 [25].

A prospective cohort study was conducted in Southern California, USA, aiming to promote the development of newborns through early mother-infant SSC during the first 3 hours after birth [10]. Of the 70% who had a vaginal birth, only 17% mother-infant dyads had SSC within the first hour, and 60% mother-infant dyads had SSC within three hours [10]. In Mexico, a recent RCT evaluated the impact of simulation and team-training program (PRONTO) on the performance of evidenced-based practice in normal birth [24]. In this RCT, the data collection was undertaken at four time points: at baseline, 4, 8 and 12 months after training. Only baseline data were included in this systematic review. The authors defined SSC as the immediate contact between mother and child after birth, and this practice was only reported to occur in 9% of births [24].

Two studies were conducted in South America, in Brazil, and both studies obtained data from “*Birth in Brazil”*, a nationwide hospital-based survey of 23,894 representative mothers and their newborns undertaken in 2011 and 2012 [37, 38]. In the study by Bladisserotto et al., SSC immediately after normal birth was reported by 34% mothers [37], and in the study by Moreira et al, by 42% of mothers after birth [38]. Although the data for both studies were from the same survey, the percentages of SSC were slightly different and so were the sample sizes (4,145 vs. 9,082). The reason behind these differences are likely due to the application of inclusion criteria in the study by Bladisserotto et al., which included postpartum women classified as low risk during pregnancy, who experienced either spontaneous or induced labor and whose birth had occurred in the Southeast region of Brazil [37].

#### South-East Asia Region

In the South-East Asia Region, four studies were conducted in Bangladesh, India, Nepal, and Sri Lanka. The study conducted in Sri Lanka reported the highest rate of SSC in this region at 50% [39]. The study evaluated the effectiveness of a training program aimed at improving the practice of essential newborn care, and half of the women interviewed before the start of the training program reported that they had SSC after birth [39].A multisite study in Bangladesh, Eastern India and Nepal aimed to understand trends in birth care practices [40]. The practice of SSC was estimated to be 30% in Bangladesh, 15% in Eastern India, and no SSC data was available from Nepal [40]. The proportion of SSC reported in Eastern India [40] was similar to the proportion of SSC reported in another Indian study at 15% [41]. Both studies lacked a clear definition of SSC practice.

#### African Region

In Africa, two studies included in the review were from Tanzania and Ethiopia. The Tanzanian study had a large sample size of 22,243 women, however, the authors did not provide a definition for the practice of SSC [42]. Less than 1% of Tanzanian women reported SSC [42], the lowest percentage amongst all studies included in this review. The proportion of women in Ethiopia who had SSC after normal birth was also low at 9% [13].

#### Eastern Mediterranean Region

The only countries reported in this review for this region were from Tunisia and Yemen. The authors of a cross-sectional study in Tunisia interviewed 354 women attending primary health centres for their child’s 6 months vaccination [43]. More than half of the women interviewed (64%) reported that they had SSC after birth. In Yemen, only 8% of women reported SSC. These studies from Tunisia and Yemen lacked clear definition of SSC and were based on small samples.

### Secondary outcomes: Starting time of skin-to-skin

The starting time of SSC was documented in 14 studies. Table 3 summarises the SSC starting time for these studies. The information is summarised according to Agudelo et al. categorisation of the time of initiation of SSC [46]: “*At birth or immediately*” when contact is made within the first minute of birth; *“Very early”* when contact made within the first 30 to 40 minutes after birth and after the mediate and immediate neonatal adaptation interventions have been carried out; and *“Early”* at any time between the first hour and 24 hours of life [46]. In this review, the “*Very early*” category extended to 60 minutes. The starting time for SSC within the 14 studies ranged from the first minute to 29 minutes. Several studies reported that SSC practice started immediately from the first minutes [14, 18, 20, 24, 29, 39]. Other studies reported that SSC was initiated within the first five minutes [23, 26] or at an average of nine minutes after birth [16]. Most of studies that reported the starting time of SSC also defined SSC and measured SSC immediately or within five minutes. None of the 14 studies indicated that SSC began after the first hour.

**Table 3:** Timing of starting skin-to-skin contact as reported in included studies (alphabetical) by author/s name.

Author/s	Country	Sample N	Immediate within first min	Very early within the first 60 min	Early at any time after 1st hour to 24h
**Bramson et al (2010) [10]**	USA	21,842	4.9%		
**Callendret et al (2015) [29]**	France	942	64.9%		
**Chalmers et al (2010) [23]**	Canada	5,600	Immediate or within 5 min = 85.7%		
**Chiou et al (2014) [14]**	Taiwan	7,828	63.8%		
**Fritz et al (2017) [24]**	Mexico	323	8.9%		
**Gubler et al (2013) [27]**	Switzerland	1,893	< 5 min = 58.5%		
**Haiek (2012) [25]**	Canada	150		5 min = 99%	
**Hakala et al (2017) [15]**	Finland	111		5–21 min = 62%	
**Kalmakoff et al (2017) [26]**	New Zealand	1,530	< 5 min = 62.8%, > 5 min = 37.2%		
**Kim (2016) [16]**	South Korea	241	1–29 min = 76.3%		
**Redshow et al (2014) [12]**	Australia	3,189	< 5 min 95.5%		
**Saxton et al (2015) [18]**	Australia	7,548	94.5%		
**Senarath et al (2007) [39]**	Sri Lanka	367	50.4%		
**Suarez-Cortes et al (2015) [20]**	Spain	9,303	Immediate 27.4%		

### Secondary outcomes: Duration of skin-to-skin contact

The duration of SSC was documented in only eight of the included studies. Table 4 presents the duration of skin-to-skin contact as reported in these studies. Most of the studies reported that the practice of SSC lasted at least for the first hour. Five studies indicated that the practice of SSC continued for two hours [10, 16, 23, 26, 27], and three studies measured the practice up to three hours after birth [10, 23, 27]. All studies that reported the duration of SSC showed that the practice of SSC was high immediately after birth and then the percentage gradually reduced except for two studies undertaken in USA and New Zealand. In the American study, 60% of the women reported they had SSC after the first hour to three hours post-birth. Across the eight studies, the duration of SSC practices was reported to be between less than five minutes and three hours.

**Table 4:** Duration of skin-to-skin contact as reported in included studies (alphabetical) by author/s name.

Author/s	Country	Sample Size	SSC practice duration per minutes							
			<5	5	10	15	30	60	120	180
**Bramson et al (2010) [10]**	USA	21,842	4.9%				12.4%		60.4%	
**Chalmers et al (2010) [23]**	Canada	5,357	85.7%		10.5%			2.2%	0.2%	
**Gubler et al (2013) [27]**	Switzerland	1,893	58.5%		38.1%				3.5%	
**Haiek (2012) [25]**	Canada	150	66%							
**Hakala et al (2017) [15]**	Finland	111		62%						
**Kalmakoff et al (2017) [26]**	New Zealand	1,530	29%					40.1%	30.9%	
**Kim (2016) [16]**	South Korea	241	76.3%							
**Redshaw et al (2014) [12]**	Australia	3,189	94.5			61.1%				

**Fig 2:**
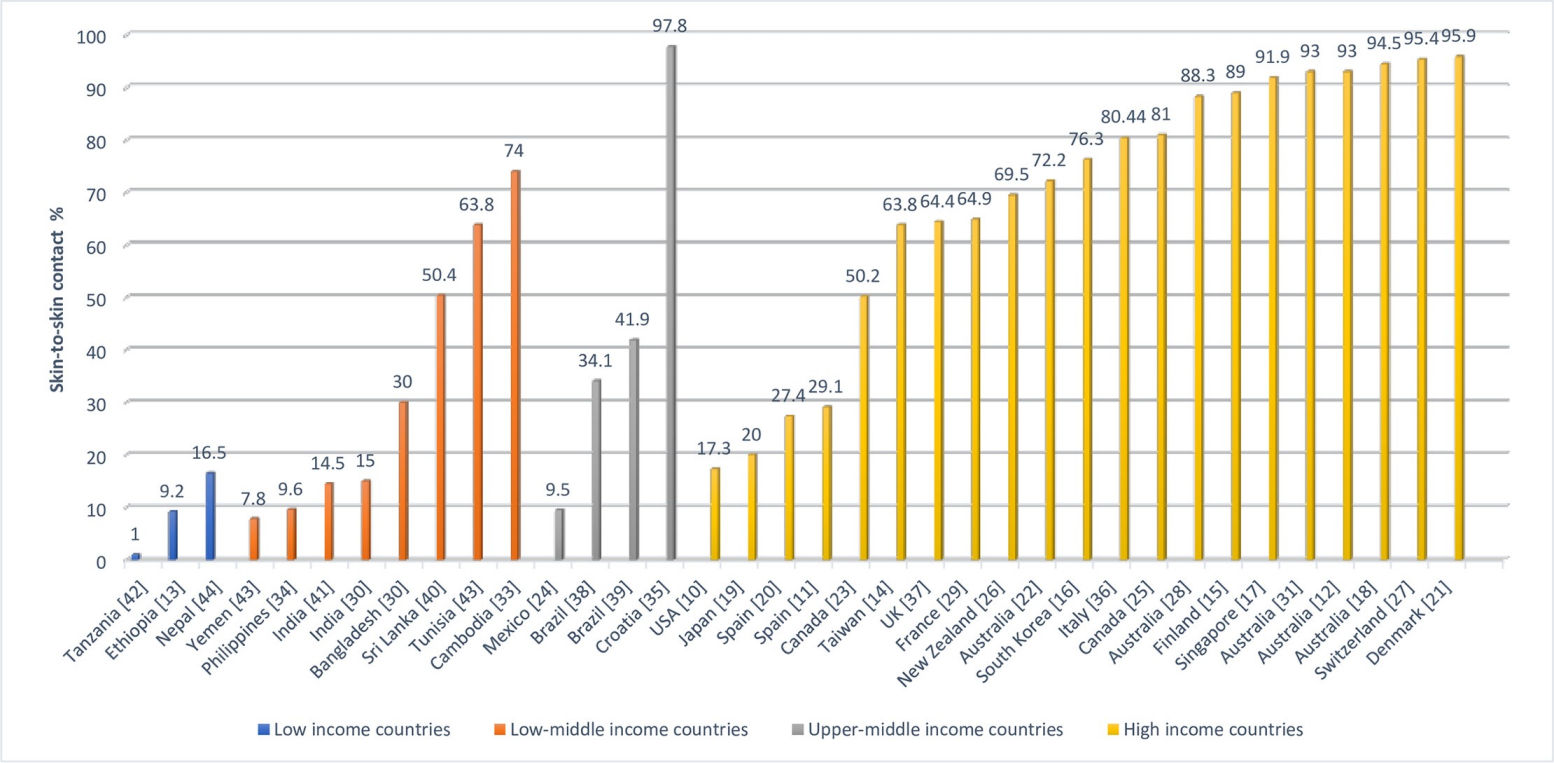
The prevalence of skin-to-skin contact for the first hour after normal birth based on World Bank classification by income.

The global prevalence of SSC based on World Bank classification by country income level is presented in Fig 2, and grouped as following: high-income countries (n = 15), upper-middle income countries (n = 3), low-middle income countries (n = 7) and low-income countries (n = 3). The practice of SSC was relatively high among high-income countries and reached a high of 96% in Denmark [21]. At the same time, high-income countries such as Japan and Spain indicated low levels of SSC practice after normal birth with 20% and 29% respectively [19, 20]. Among the upper-middle income countries, the trend of SSC ranged between 9% in Mexico and up to 98% in Croatia. The study conducted in Croatia reported the highest rate of SSC among all countries with almost 98%. In low-middle income countries the practice of SSC varied, and a range between 8% to 74% was documented across studies. Low-income countries including Tanzania, Ethiopia and Nepal had reported beneath 20% the practice of SSC after normal birth.

## Discussion

This study is the first systematic review to our knowledge that attempts to report the worldwide prevalence of SSC after normal birth, using available information from low, middle and high-income countries. We addressed several aspects of SSC practice including the proportion of SSC, starting time and duration.

In this review we found a wide range in the overall prevalence of SSC immediately after birth from a low of 1% in Tanzania [42] to a high of 98% in Croatia [34]. The disparities in our review can be explained in a number of ways. Firstly, the observed differences across countries could be attributed to the lack of agreed definition used in the included studies. The authors of the studies defined SSC in different ways, for instance, “naked baby on mother’s naked body” [25] “first contact” [22], “immediate contact between mother and newborn” [24] and “close contact”[21], which makes comparisons difficult. Furthermore, the criteria for starting SSC, baby position, and duration of SSC were not well described.

The WHO has released a new guideline “Protecting, promoting and supporting breastfeeding in facilities providing maternity and newborn services” in 2017 [1]. In this guideline, WHO provided a definition of SSC: “when the infant is placed prone on the mother’s abdomen or chest in direct ventral-to-ventral skin-to-skin contact. Immediate skin-to-skin contact is done immediately after delivery, less than 10 minutes after birth. Early skin-to-skin contact was defined as beginning any time from delivery to 23 hours after birth. Skin-to-skin contact should be uninterrupted for at least 60 minutes” p.5 [1]. Prior to this definition by the WHO, there was no standardised definition of SSC, and this may be one contributing factor to the wide disparities in the definitions used across studies.

The heterogeneity of definition is somewhat similar to what was reported in a systematic review of Kangroo Mother Care (KMC) in different settings, where SSC was the core component of KMC [47]. The authors highlighted the need to standadise the definitons of KMC and SSC, and to differentate between these practices [47]. Thus, it is highly recommended to standadise the defnintion of SSC in all future studies according to the WHO definition [1].

Secondly, methodological diffrences among studies including different study designs may also have contributed to the variation in documented SSC. Observational studies appears to have low rates of SSC; from 9% in the Philippines to 29% in Spain [11, 33], whereas cross-sectional studies and retrospective studies reported higher level of SSC practice; in Singapore and Australia 92% and 95%, respectively [17, 18]. Therefore, threre is a need for future observational studies because of its valid design reflecting the actual practcie. A novel algorithm was recently published that systematically analyses and measures the practice of SSC in relation to the best practice of immediate, continuous, and uninterrupted SSC [48]. This algorithm, if effectively adopted would improve the practice of SSC and enhance the development of strategies to implement the practice of SSC at the WHO standard of immediate, continuous and uninterrupted SSC [49].

Thirdly, the timing of data collection is also important, as there is a risk for recall bias if the mother is not surveyed soon after birth when she have better recollection of the birth experience. In Canada, two studies collected data about SSC at two different times; one study interviewed women at two months after birth, with a reported 81% SSC [25], while the other interviewed women five to ten months after birth, with a reported rate of SSC of 50% [23]. Even though the studies were based on different samples, these two figures indicate that timing of data collection could possibly influence the reported rate of SSC. Furthermore, there was a lack of description across all studies with regards to who was actually reporting the practice of SSC the mother herself, hospital staff or researcher. It is important to identify the reporting person to increase study validity.

Fourthly, another possible explanation for the variation of reported SCC is the level of country income. Most studies from high-income countries such as, Denmark, Switzerland, Australia, Finland and Singapore, and upper-middle income countries as Croatia, reported high levels of SSC practice (Fig 2)Some high-income countries reported low practice of SSC such as Japan with 20% [19] and Spain with an average of 29% [11, 20]. In South California, USA, where one study indicated that only 17% mother-infant dyads practiced SSC within the first hour after normal birth however, the practice was significantly higher within three hours after birth: 60%. A national survey of 10,000 women was published in the UK in 2014 [50]. This study which was not included in our systematic review as it wasnot identified in our search strategy, however it reported that 85% of women experienced SSC, which is considerably higher than the 64% identified in our only UK study of 111 women. This illustrates that conclusions about SSC practices from the studies included in our review cannot be made as they were not based on national representative samples.

Another factor to consider is change over time, however, when looking at the average SSC rate per year, there are no indications of change over the ten year period (2007 to 2017), and thus, the data do not support that improvements have been made in relation to SSC practices worldwide. The most recent studies published in 2016 and 2017 still reported a low proportion of SSC practices in Brazil (34%) [37], Ethiopia (8%) [13], and Mexcio (10%) [24].

Finally, despite the lack of common definitions and variety of study designs in the included studies, we can conclude that the level of SSC differs greatly across the globe. The fact that only three of the 35 included studies, representing 28 countries, focused specifically on assessing the practice of SSC indicates the need for more research on this practice. According to the 2017 WHO guideline, there is a need for more research on the time of initiation of SSC and the long term effects on infant neurodevelopment and health outcomes [1].

### What did we find in this systematic review?

- Among the included studies there was a lack of agreed definition about skin-to-skin contact.
- There was a wide variation of the actual practice among countries from 2007 and 2017 and no change was identified over time.
- Few studies were conducted with the primary aim to measure the practice of SSC worldwide.
- There is a lack of studies about SSC from low income countries.
- Few studies reported starting time and duration of SSC practice after normal birth.

### Strength and limitation

This review had a broad scope and included studies were based on different methodologies, including RCTs, secondary analyses, and routine data to extract of SSC outcomes regardless of the study aim. This enabled us to report the practice of SSC from countries distributed across the WHO world regions. We limited the search to studies published within the last ten years in order to reflect the current practice of SSC. The data extraction and selection of studies was precise and based on a thorough quality assessment to eliminate low quality studies.

Our review also has number of limitations. Although we identified a considerable number of studies for this systematic review, most were not designed to measure the prevalence of SSC and were not based on national samples. Generalizability of the result is therefore limited, particularly due to the fact that many studies were based on small samples not representative of the country. Other major limitations were the lack of a common SSC definition and heterogeneity of study design.

### Recommendations

We highly recommended a standardised use of the definition of SSC according to the suggestion by the WHO [1] for all studies focused on the practice of SSC. More robust studies or observational studies about the practice after normal birth are needed to estimate the prevalence of SSC. Future studies and guidelines to improve immediate, continuous and uninterrupted SSC should include a standardised set of indicators and measurement tools that document SSC starting time and duration. A tool that suggests the appropriate time to ask questions about the experience about SSC, or an observation checklist tool that can assist nurses or midwives when observing the practice would also be helpful. Ideally data should be collected through observation of the birth or a self-reported questionnaire for the mother shortly after birth. There is a need to translate the research into practice by evaluating interventions to improve SSC. More high-quality research is needed in relation to the practice and implementation of SSC. Inclusion of SSC in the governmental perinatal data collections, including initiation time and duration, would allow for a more accurate estimation of the prevalence of SSC at population levels.

## Conclusion

There is a strong evidence to support the benefits of skin-to-skin contact after normal birth and this review attempted to describe the available data about the practice of SSC worldwide. The study indicates that the practice of SSC varies substantially across the world, from a reported 1% to 98%, also with varying starting times and durations. However, limitations including lack of nationally representative studies and common definitions of SSC, which prevents us from drawing firm conclusions. Future studies need to standardise the definition of SSC to enable measurement of the prevalence of immediate, continuous and uninterrupted SSC and facilitate the implementation of this important practice.

## Supporting information

S1 Table CINAHL search strategy. (DOCX)

S2 Table Joanna Briggs Institute (JBI) assessment of methodological quality tool check list. (DOCX)

S3 Table PRISMA checklist. (DOC)
