# Supplementary material for: Worldwide prevalence of mother-infant skin-to-skin contact after vaginal birth: A systematic review
Source: PLoS One. 2018 Oct 31;13(10):e0205696. doi: 10.1371/journal.pone.0205696 (PMC6209188; doi:10.1371/journal.pone.0205696)
Supplement: S1 Table — (DOCX) [file pone.0205696.s001.docx]

**S1 CINAHL Search strategy**

|  |  |
| --- | --- |
| Steps |  |
|  | ("Kangaroo Care" or "skin to skin contact" or "breastfeeding initiation" or "breast crawl" or "maternal infant contact" or "maternal newborn contact" or "baby friendly hospital initiative" or "ten steps for successful breastfeeding").mp. [mp=title, abstract, original title, name of substance word, subject heading word, keyword heading word, protocol supplementary concept word, rare disease supplementary concept word, unique identifier, synonyms] |
|  | Limiters - Peer Reviewed, Published Date 2007/01/01-2017/10/18. |
|  | Search modes - Boolean/Phrase |
|  | Interface - EBSCOhost research Database |
|  | Results - 916 |
