# Supplementary material for: Worldwide prevalence of mother-infant skin-to-skin contact after vaginal birth: A systematic review
Source: PLoS One. 2018 Oct 31;13(10):e0205696. doi: 10.1371/journal.pone.0205696 (PMC6209188; doi:10.1371/journal.pone.0205696)
Supplement: S2 Table — (DOCX) [file pone.0205696.s002.docx]

**S2 .Table Joanna Briggs Institute Assessment of methodological quality**

| Study | Q1 | Q2 | Q3 | Q4 | Q5 | Q6 | Q7 | Q8 | Q9/RR% | Comment |
| --- | --- | --- | --- | --- | --- | --- | --- | --- | --- | --- |
| 1. **Andersson, Flems, and Kesmodel (2016) [21]** | Y | Y | Y/274253 | Y | Y | Y | U | Y | Y/90 | Included |
| 1. **Baldisserotto, Theme Filha, and da Gama (2016) [38]** | Y | Y | Y/4102 | Y | Y | Y | Y | Y | Y/NG | Included |
| 1. **Bouanene et al., (2010) [47]** | Y | Y | Y/354 | Y | Y | Y | Y | Y | N/NG | Included |
| 1. **Bramson et al. (2010) [10]** | Y | Y | Y/21842 | Y | Y | Y | Y | Y | Y | Included |
| 1. **Brodribb, Kruske, and Miller (2013) [21]** | Y | Y | Y/6752 | Y | Y | Y | Y | Y | Y/35.8 | Included |
| 1. **Callaghan et al., (2016) [13]** | Y | Y | Y/215 | Y | Y | Y | Y | Y | U/NG | Included |
| 1. **Callendret et al. (2015) [34]** | Y | Y | Y/993 | Y | Y | U | Y | Y | Y/NG | Included |
| 1. **Cederfeldt, Carlsson, Begley, and Berg (2016) [45]** | Y | Y | Y/164 | Y | U | Y | Y | Y | N/NG | Included |
| 1. **Chalmers et al. (2010) [37]** | Y | N | Y/5,357 | N | Y | Y | Y | Y | Y/78 | Included |
| 1. **Chiou et al., (2014) [14]** | Y | Y | Y/12455 | Y | Y | Y | Y | Y | Y/NG | Included |
| 1. **Crowe et al., (2015) [41]** | Y | Y | Y/ B=27533  I=8939 | Y | Y | U | Y | Y | U/NG | Included |
| 1. **Dauletyarova et al., (2016)** | Y | Y | Y/872 | Y | U | U | U | Y | N | Excluded |
| 1. **de Graft-Johnson et al. (2017)** | Y | Y | Y/2377 | U | Y | U | U | Y | N | Excluded |
| 1. **Fritz et al., (2017) [24]** | Y | Y | Y/641 | Y | Y | Y | N | Y | U/NG | included |
| 1. **Gubler, Krähenmann, Roos, Zimmermann, and Ochsenbein-Kölble (2013) [36]** | Y | Y | Y/1893 | Y | Y | Y | Y | Y | NA | Included |
| 1. **Haiek (2012) [25]** | U | Y | Y/150 | Y | Y | U | U | Y | Y88 | Included |
| 1. **Hakala et al. (2017) [15]** | Y | Y | U/111 | Y | Y | Y | Y | Y | Y59 | Included |
| 1. **Hongo, Nanishi, Shibanuma, and Jimba (2015) [19]** | Y | Y | Y/363 | Y | Y | Y | Y | Y | Y/60 | Included |
| 1. **Kalmakoff, Gray, and Baddock (2017) [26]** | Y | Y | Y/1530 | Y | Y | Y | Y | Y | NA | Included |
| 1. **Keemer (2013) [27]** | Y | Y | Y/128 | Y | Y | U | Y | Y | Y/56.9 | Included |
| 1. **Kempe et al., (2010) [48]** | Y | Y | Y/220 | Y | Y | U | U | Y | Y/100% | Included |
| 1. **Kim (2016) [16]** | Y | Y | Y/366 | U | N | Y | Y | Y | Y/20 | Included |
| 1. **Lau et al. (2017) [17]** | Y | Y | Y/915 | Y | Y | Y | Y | Y | Y/90.4 | Included |
| 1. **Lauria, Spinelli, and Grandolfo (2016) [33]** | Y | Y | Y/6942 | U | Y | U | Y | Y | U | Included |
| 1. **Macfarlane, Rocca-Ihenacho, and Turner (2014) [35]** | Y | Y | Y/166 | Y | Y | Y | Y | Y | Y/66.4 | Included |
| 1. **Martinez-Galiano and Delgado-Rodriguez (2014) [46]** | Y | Y | Y/520 | Y | U | Y | Y | Y | U | Included |
| 1. **Moreira et al. (2014) [39]** | Y | Y | Y/18639 | Y | Y | U | U | Y | Y/NG | Included |
| 1. **Ogbo et al. (2016) [28]** | Y | Y | Y/17564 | Y | Y | Y | Y | Y | NA | Included |
| 1. **Penfold et al. (2010) [43]** | Y | Y | Y/22,243 | Y | U | Y | U | U | Y/92% | Included |
| 1. **Pereira, Fonseca Vde, Couto de Oliveira, Souza, and Reis de Mello (2013)** | Y | Y | Y/403 | Y | N | U | U | Y | Y | Excluded |
| 1. **Pierro, Abulaimoun, Roth, and Blau (2016)** | Y | Y | Y/712 | Y | U | U | U | U | Y | Excluded |
| 1. **Redshaw, Hennegan, and Kruske (2014) [12]** | Y | Y | Y/4574 | Y | Y | Y | Y | Y | Y/30.4 | Included |
| 1. **Salvador et al., (2016)** | U | U | Y/222 | N | U | U | U | N | U | Excluded |
| 1. **Sandin-Bojo et al., (2012) [30]** | U | Y | U/177 | Y | Y | Y | Y | Y | Y/NG | Included |
| 1. **Saxton, Fahy, Rolfe, Skinner, and Hastie (2015) [18]** | Y | Y | Y/7548 | Y | U | N | Y | Y | N/A | Included |
| 1. **Senarath, Fernando, and Rodrigo (2007) [40]** | Y | Y | Y/466 | U | Y | U | Y | Y | Y/NG | Included |
| 1. **Sobel, Silvestre, Mantaring, Oliveros, and Nyunt (2011) [31]** | Y | Y | Y/481 | U | Y | Y | Y | Y | N/NG | Included |
| 1. **Suarez-Cortes et al., (2015) [20]** | Y | Y | Y/9303 | U | Y | Y | Y | Y | U/NG | Included |
| 1. **Suzuki (2013)** | Y | U | Y470 | U | U | U | U | U | U | Excluded |
| 1. **Upadhyay, Rai, and Anand (2012) [42]** | Y | Y | Y/415 | Y | U | Y | Y | Y | U/NG | Included |
| 1. **Zakarija-Grkovic, Boban, Jankovic, Cuze, and Burmaz (2017) [32]** | Y | Y | Y/733 | Y | Y | Y | Y | Y | U/NG | Included |

B: Bangladesh, I: India, N: NO, NA: Not applicable NG: Not given Y: Yes, U: Unclear, and *Percentage included if it was available in the article.
